# Molecular Interplay of PARN and Telomerase: Tail Modifiers and Disease Implications

**DOI:** 10.1002/wrna.70049

**Published:** 2026-06-23

**Authors:** Sujitha Felicitus, Shalon Suzanne Pinto, Sai Samanvitha Madagundapalli Ramakrishna, Dechamma Pandyanada Nanjappa, Gunimala Chakraborty, Anirban Chakraborty

**Affiliations:** ^1^ Department of Molecular Genetics and Cancer Nitte (Deemed to be University), Nitte University Centre for Science Education and Research (NUCSER) Mangalore India; ^2^ Department of Medical Oncology Nitte (Deemed to be University), KS Hegde Medical Academy Mangalore India

**Keywords:** aging, cancer, genomic instability, PARN, RNA stability, telomerase, telomere, telomere biology disorders (TBDs), TERC, TP53

## Abstract

Poly(A)‐specific ribonuclease (PARN) and telomerase are two indispensable tail‐modifying enzymes that are required in two critical cellular processes: the control of the rate of RNA stability and maintenance of telomere integrity, respectively. Pathogenic variations in genes that code for PARN and telomerase complex enzymes have been implicated in several rare genetic diseases, including bone marrow failure syndromes, telomere biology‐related disorders, and neoplastic ailments. This is further exemplified by defects in p53 signaling, which not only exacerbate the effects of telomere shortening but also negatively regulate PARN activity, thereby promoting cancer development and accelerated aging. This review examines the molecular interactions between PARN and telomerase, as well as their implications in disease development, with a focus on emerging therapeutic strategies that target the pathways regulated by these two enzymes.

## Graphical abstract

Schematic representation of the molecular interplay between PARN, telomerase‐associated, and the involvement of p53 in regulating telomere maintenance and genome stability. The top panel shows how normal PARN levels are involved in regulating p53 levels and balanced telomerase activity through their regulatory interaction with TERC and TERRA, thus contributing to genomic stability. Whereas the uncontrolled role of the telomere maintenance mechanism with the elevation in PARN levels can lead to abnormally elevated telomerase function, increasing cancer incidence. The bottom panel illustrates deregulated telomere maintenance and PARN deficiency resulting in impaired TERC maturation due to PAPD5‐mediated polyadenylation resulting in telomere shortening, p53 activation and onset of telomere biology disorders. Persistent telomere dysfunction over time leads to genomic instability, causing exposed ends of chromosomes to undergo end‐to‐end fusion and increasing the risk of cancer, as seen in TBD patients. This image was created using Adobe Illustrator.

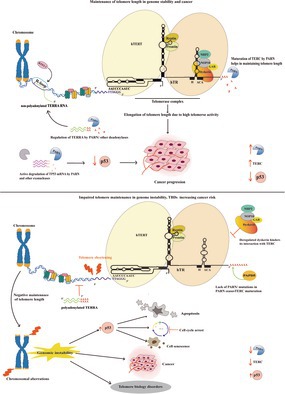

## 1 Introduction

The discovery of telomeres by Muller (1940) in Drosophila and by McClintock (1941) in corn led to the basis of understanding the limited replicative potential of cells, which was reported by Gall (1995), Hayflick and Moorhead (1961), and Zakian (2012). This shift in understanding increased the focus on investigating the role of telomeres and their tail‐modifying ribonucleoprotein enzyme, telomerase, in safeguarding the chromosome ends, a function demonstrated by Blackburn et al. (2009), which fetched them the Nobel Prize in Physiology and Medicine. This discovery encouraged extensive research into telomere biology, with significant implications in cancer, aging, and other human diseases.

The initial notion about telomere length was that it shortens with age, which corresponds to the replicative potential of cells. However, an exception to this concept arose when the telomeres appeared to be growing with advancing age (Haussmann and Mauck 2008). Besides lifespan, telomere length was also found to be influenced by an organism's size (Risques and Promislow 2018). This observation led to the suggestion that body size, rather than life expectancy, may correspond with telomerase activity, which further may have helped in maintaining their telomere length (Gorbunova and Seluanov 2009). Another interesting finding from Fohringer et al. (2022) revealed that a moose living in stable environmental areas had longer telomeres than those inhabiting unstable environments, emphasizing the impact of environmental factors on telomere dynamics in mammals. Subsequent research in this field revealed additional influencing factors that determine telomere length. It became evident at the molecular level that while telomeres protect the chromosome ends from replicative senescence and DNA damage response, critically short telomeres result in chromosomal fusions, promoting genomic instability, leading to telomeropathies and an increased cancer risk. Whereas abnormally long telomeres permit uncontrolled cell division through telomerase reactivation, thereby also contributing to tumorigenesis. Therefore, maintaining the optimal or “just right” telomere length as explained in the “Goldilocks” principle of telomere biology by Savage (2024), is important in maintaining genomic stability and proper physiological functions essential for sustaining cell division throughout life (Mitchell et al. 1999). Although this review focuses on telomerase, it is important to note that telomere length dysregulation can arise from pathogenic variants in both telomerase coding genes, that is, telomerase reverse transcriptase (*TERT*) and telomerase RNA component (*TERC*) and several other genes that code for factors in telomere biology such as Dyskerin Peudouridine Synthase (*DKC1*), Poly(A)‐specific ribonuclease (*PARN*), nucleolar protein 10 (*NOP10*), Non‐histone protein 2 (*NHP2*), regulator of telomere elongation helicase 1 (*RTEL1*) and telomeric repeat binding factor 1 (*TERF1*). Together, mutations in these genes disrupt the telomere homeostasis, which has been implicated in a spectrum of rare hereditary diseases known as telomere biology disorders (TBDs) that are characterized by early cell aging and a myriad of health problems, with a high predisposition to cancer (Savage and Bertuch 2010; Niewisch et al. 2023; Giri et al. 2020).

An intriguing but exciting domain was revealed when pathogenic variations in *PARN* were discovered in TBDs (Moon et al. 2015; Tummala et al. 2015; Dhanraj et al. 2015; Burris et al. 2016), indicating that the functions of these two critical enzymes could be interlinked through a common pathway. It has long been recognized that PARN's basic role is to deadenylate mRNAs, which is the first step in deadenylation‐dependent mRNA decay. The conventional role of this multifunctional deadenylating enzyme was discussed in our previously published review titled “PARN: More than just ‘mRNA stock clearing’” (Nanjappa et al. 2021). In this review, the focus is on illustrating the unique role of PARN in telomere maintenance and its relevance in TBDs.

## 2 Traditional Role of PARN in RNA Stability Through Poly(A) Tail Maintenance

**FIGURE 1:**
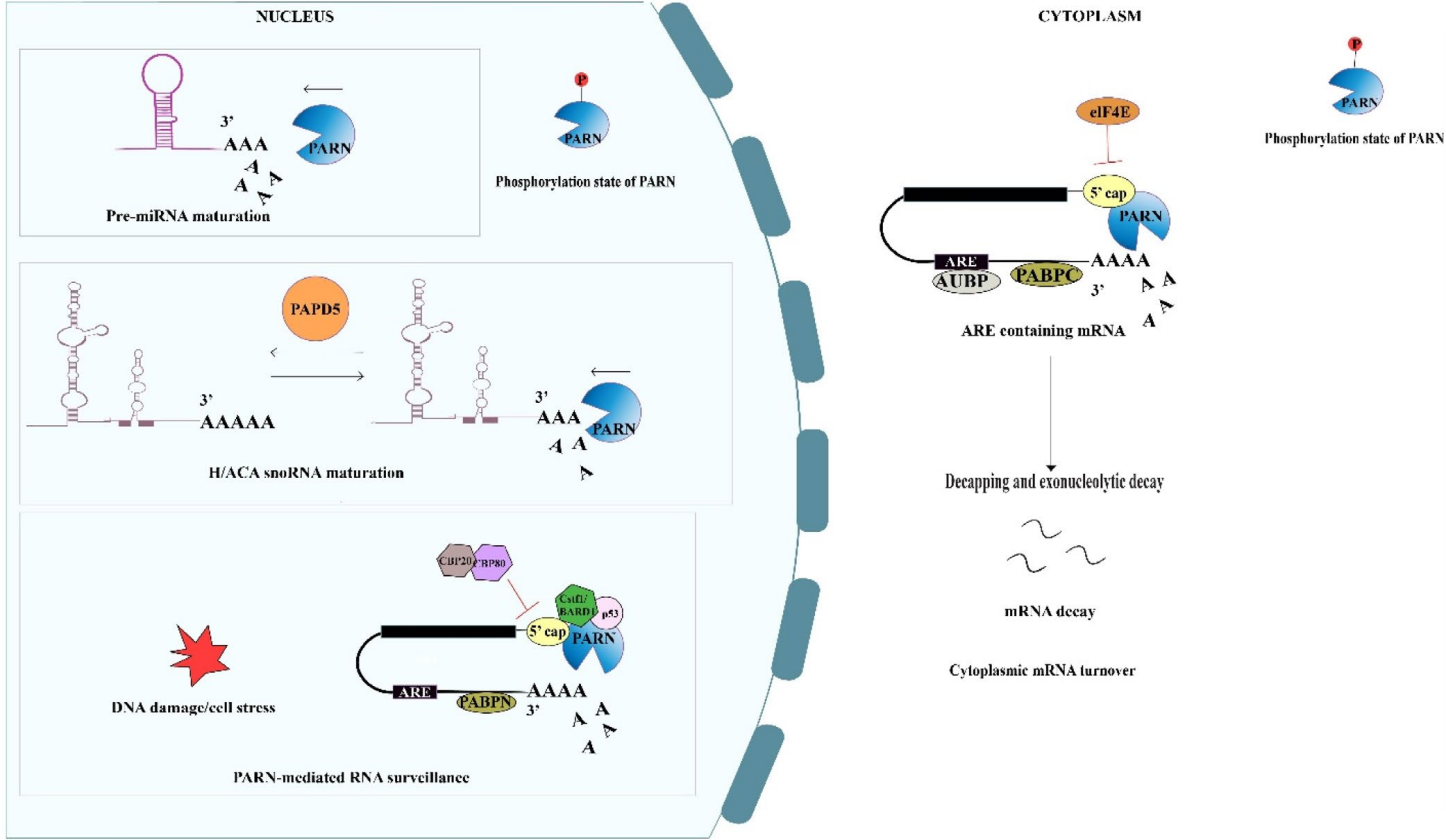
Schematic representation of the biochemical pathways of PARN‐mediated deadenylation and its major RNA substrates. Poly(A)‐specific ribonuclease (PARN) is a 3′‐5′ exoribonuclease that regulates RNA metabolism through the controlled shortening of poly(A) tails, thereby influencing RNA maturation, quality control, and turnover. In the nucleus, PARN participates in the maturation and processing of noncoding RNAs, including H/ACA box snoRNAs, telomerase RNA component (TERC), and pre‐miRNAs. PAPD5‐mediated oligoadenylation of these RNA substrates is counterbalanced by PARN‐mediated deadenylation, thus maintaining RNA stability and proper ribonucleoprotein assembly, whereas excessive trimming can promote RNA degradation. PARN also contributes to nuclear RNA surveillance by facilitating the degradation of aberrant transcripts generated during cellular stress, DNA damage, defective ribonucleoprotein assembly, or faulty RNA processing. In addition, PARN activity is regulated by its phosphorylation state. PARN can compete with the nuclear cap binding complex (CBC), particularly CBP80, for access to the 5′ cap structure. The dissociation of CBP80 during cellular stress promotes PARN association with the 5′ cap, enabling the 3′ end deadenylation‐dependent decay of mRNA. In the cytoplasm, PARN regulates mRNA turnover by deadenylating transcripts containing long poly (A) tails and specific cis‐regulatory elements, including AU‐rich elements (AREs), thereby contributing to translational repression and mRNA decay. PARN activity is further modulated by cellular stress responses and posttranslational modifications such as phosphorylation, allowing dynamic regulation of RNA stability. *TP53*, c‐*MYC*, c‐*FOS*, and *TNF‐α* are among the known ARE‐containing transcripts, making them susceptible to PARN‐mediated deadenylation. Consequently, PARN contributes to the regulation of *TP53* mRNA under both stress and non‐stress conditions. Similar to its nuclear role, dissociation of elF4E from the cytoplasmic 5′ cap facilitates PARN recruitment to the mRNA 5′ cap, promoting 3′ end deadenylation and translational repression. Conversely, PARN activity is inhibited by cap‐binding complexes (CBCs) and poly(A)‐binding proteins (PABPs), including nuclear PABPN and cytoplasmic PABPC, which protect mRNAs from premature deadenylation and degradation. Together, these nuclear and cytoplasmic functions establish PARN as one of the major regulators of RNA homeostasis, coordinating the maturation, surveillance, and degradation of both coding and noncoding RNAs. This image was created using Adobe Illustrator.

Evidence from various studies suggests that the poly (A) tail of RNA, including both mRNAs and noncoding RNAs, undergoes dynamic alterations in length both within the nucleus and postnuclear transport, a phenomenon that is conserved across different species and is regulated in a transcript‐specific manner, thereby prompting an investigation into its potential role as a molecular ruler for RNA stability (Dworkin and Dworkin‐Rastl 1985; Salles et al. 1994; Verrotti et al. 1996). The 3′ end of the eukaryotic nascent RNA transcripts, including pre‐mRNAs, certain noncoding RNAs such as telomerase RNA precursors, receives adenosine residues through a co‐transcriptional polyadenylation step during transcription by RNA polymerase II. Subsequent modulation of poly (A) tail length occurs both co‐transcriptionally and posttranscriptionally and is influenced by transcript‐specific regulatory factors such as sequences in the 3′ UTR regions, codon optimality, RNA binding proteins, and cellular state (Daniel Jr 1997). In mammalian cells, the 3′ untranslated region of RNAs has special cis‐acting areas of AU‐rich elements (AREs) Figure 1, which aid in regulating the stability and pace of RNA decay. ARE‐binding proteins (ARE‐BPs) that recognize and bind to these elements can either stabilize or destabilize the transcript, with the final outcome determined by the competition between the stabilizing and the destabilizing factors that interact with AREs. If the outcome is destabilization of the transcript, it leads to the removal of the poly (A) tail residues, thereby initiating the deadenylation‐dependent RNA degradation (van Hoof and Parker 2002; Gherzi et al. 2004; Ford et al. 1997). Among the many deadenylases, an interesting one is poly (A)‐specific ribonuclease (PARN), which is a multifunctional 3′‐5′ exonuclease and also the only deadenylase known to interact both with the 5′ cap structure (Gao et al. 2000; Martınez et al. 2000; Dehlin et al. 2000) and the adenosine residues at the 3′ end of mature RNAs (Martînez et al. 2001). PARN, like any other RNA binding proteins, has three main domains (Wu et al. 2005; Monecke et al. 2008) that play a major role in their recognition and binding of RNA substrates. The RNA recognition motif domain allows the binding of a poly (A) tail to PARN, and the strongest binding happens when there is a presence of 20 adenosine residues. This domain also has a separate site for the 5′ cap binding that promotes its deadenylating activity (Nilsson et al. 2007). The next is the nuclease domain, which overlaps the active site and the cap‐binding site and allows it to bind with higher affinity when 5′ cap structure is present in cis. The third is the R3H domain that shows more relaxed specificity to the poly (A) tail (Virtanen et al. 2010; Henriksson et al. 2010). Although a number of deadenylases have been reported, PARN is suggested to be the predominant one in mammals (Gao et al. 2001).

PARN is known to distinguish between different RNA species, both at transcriptional and posttranscriptional levels. In the nucleus, modifications of RNA happen during the transcription not only to protect them from decay but also to help cellular machineries to distinguish them as different from those that are marked for translation/degradation.

### 2.1 PARN‐Mediated Deadenylation—The Co‐Transcriptional Regulators

In the nucleus, the inhibition of PARN‐mediated deadenylation is carried out by CBP80, which physically binds to one of the subunits of PARN and prevents its interaction with the 5′ cap of mRNA (Martınez et al. 2000; Wu et al. 2005; Balatsos et al. 2009). However, during DNA damage response (DDR), this interaction is perturbed by the presence of cleavage‐stimulating factor subunit 1 (Cstf1) and BARD1/BRCA1 complex, which dissociates the binding of CBP80 to PARN, thus promoting deadenylation. Interestingly, p53 was also shown to promote PARN‐mediated deadenylation during cellular stress conditions by directly interacting with PARN—Cstf1/BARD1 complex through its c‐terminal domain, thus controlling gene expression during cellular stress conditions (Cevher et al. 2010; Nazeer et al. 2011; Devany et al. 2013). Intriguingly, PARN also controls *TP53* mRNA levels through its interaction with Nucleolin (NCL), which is a stress‐responsive RNA‐binding protein (RBP). During normal conditions, NCL in its phosphorylated state, binds to the N‐terminal domain (NTD) of PARN and the ARE region adjacent to the miRNA target site in the 3′ UTR of *TP53* mRNA, thus favoring miRNA‐induced mRNA decay (Devany et al. 2013; Zhang et al. 2015). In UV‐induced cellular stress, phosphorylation‐deficient NCL fails to bind to the NTD domain of PARN and the ARE of *TP53* mRNA. This allows ARE‐binding protein, human antigen R (HuR) to bind to and stabilize the *TP53* mRNA, thereby enhancing its translation (Zhang et al. 2018). Similar involvement of PARN and p53 was observed with the Tau protein, which is a microtubule‐associated protein found in neurons of the central nervous system. During DDR, Tau forms a complex with PARN, p53, and Pin1 (peptidylprolyl isomerase), which activates PARN‐mediated deadenylation (Baquero et al. 2019).

PARN also regulates the expression of other mRNAs involved in DDR, including c‐*MYC*, c‐*FOS*, *TNF‐α* through similar mechanisms (Devany et al. 2013). Besides mRNAs, PARN has been shown to be involved in modulating miRNA levels (Lee et al. 2019). For instance, knockdown of PARN in human cells led to a reduction in levels of miR‐21‐5p, miR‐181b‐5p, and miR‐92b‐3p, with a concomitant increase in the oligoadenylation of these miRNAs, indicating a direct role of PARN for their stability (Shukla, Bjerke, et al. 2019; Shukla, Spurrier, et al. 2019; Tomasello et al. 2024). Similarly, in snoRNAs, the intermediate forms are oligoadenylated by poly (A) RNA polymerase D5 (PAPD5), which further undergo deadenylation by PARN for maturation. This process of polyadenylating/deadenylating of snoRNAs stabilizes their structure and helps in maintaining steady‐state levels of snoRNAs (Berndt et al. 2012). PARN is also essential for the maturation and homeostasis of H/ACA box snoRNAs (Berndt et al. 2012). Collectively, these findings suggest that PARN is directly or indirectly controlled by positive and negative regulators within the nucleus through its involvement in mRNA processing, surveillance, and decay, positioning itself as one of the key regulators of nuclear RNA stability.

### 2.2 PARN‐Mediated Deadenylation—The Post‐Transcriptional Regulators

In the cytoplasm, posttranscriptional regulation by PARN is mediated through the cis‐acting sequences in the 3′ UTR regions, especially the ARE regions and also the mRNP complexes (Lee et al. 2012). During translation, the 5′ cap structure of the mRNAs is bound by cytoplasmic cap‐binding proteins such as the translation initiation factors elF4E complex, which then interacts with the poly (A) binding proteins (PABPs), resulting in the formation of a stable circular mRNP that inhibits PARN activity. Transcripts with too long or too short poly (A) tails may not accommodate enough PABP to bind, thus exposing the ARE binding sites and the resultant in the activation of the deadenylase activity. The extended or shortened poly (A) tails dissociate elF4E from the cap, thereby promoting the interaction of PARN with the 5′ cap and 3′ tail, to form a loop structure which initiates the 3′–5′ mRNA decay (Gaoet al. 2000). In the absence of PABPs, CUG‐binding proteins (CUG‐BP) that interact with the AU‐rich and GU‐rich elements of the mRNAs, recruit PARN for its deadenylation activity (Moraes et al. 2006; Lee et al. 2012). In addition to the poly(A) tail length, ARE & miRNA‐target regions, and the mRNP interactions, deadenylation by PARN is also dependent on the phosphorylation states of PARN and elF4E. For instance, a study has shown that serum starvation in cells led to an increase in phosphorylation of PARN and elF4E, resulting in the dissociation of elF4E from the 5′ cap, thus allowing more efficient binding of PARN to the 3′ poly (A) tail and 5′ cap, and an increased mRNA degradation (Seal et al. 2005). In another study done in mouse myeloblasts to investigate a set of mRNAs that are dependent on PARN, it was observed that knockdown of *PARN* stabilized around 40 mRNAs (2.9%) out of 1389 mRNAs, suggesting that their deadenylation was dependent on PARN (Lee et al. 2012). One of them was zinc finger protein 36 like 2 (Zfp36l2), which has a long poly (A) tail. In the same study, it was reported that PARN KD cells migrated faster in wound healing assays, suggesting an increased expression of genes, most likely due to a reduced deadenylation. A similar observation was also reported in another study involving gene ontology and microarray results (Devany et al. 2013), implying that the genes involved in cell migration and motility may depend on PARN for their regulation. However, further confirmatory analysis needs to be carried out to conclusively prove this as a consequence of *PARN* loss of function or enhanced function of genes involved in mRNA metabolic pathways (Devany et al. 2013). However, it still remains unclear whether the choice of a deadenylase depends on the type of the RNA and whether global mRNA decay rate is regulated by specific deadenylases. Nevertheless, from the above findings, it is reasonable to say that PARN operates predominantly as a primary regulator for certain RNA substrates and a redundant regulator for bulk RNA turnover compared to the exosome complex, which are the dominant protein complex in the case of nuclear RNA turnover (Liu et al. 2014). PARN and exosome are both redundant in the cytoplasmic region of the cellular compartment, where both can cooperate to carry out the process of mRNA decay (Schoenberg and Maquat 2012). Collectively, PARN is involved in the regulation when it is an ARE‐mediated decay (Zhang et al. 2015), during oocyte maturation in *Xenopus l*arvae (Copeland and Wormington 2001), and during DNA damage response to stress (Duan et al. 2019). It is also regulated by the presence of ARE‐BP like tritetraprolin (TTP) (Lin, Duffy, and Chen 2007; Lin, Lin, et al. 2007), KH‐splicing regulatory proteins (KSRP) (Gherzi et al. 2004), and HuR (Friedersdorf 2011) and has a primary role in maturation of noncoding RNA substrates (Berndt et al. 2012) and miRNAs (Shukla, Bjerke, et al. 2019; Shukla, Spurrier, et al. 2019; Lee et al. 2019). Like telomeres, PARN‐mediated deadenylation generates transcripts with optimal poly (A) tail lengths, producing a “just right” state, which is determined by the identity of the transcript and cellular conditions that occur both in co‐transcriptional and posttranscriptional as discussed above. Studies by Kim and Richter (2006); Bachvarova (1992 ) demonstrated that PARN acts as a processive deadenylase with high affinity for poly (A). Its activity is regulated by a complex of factors as discussed above to maintain a poly(A) tail that is short enough to avoid degradation and premature translation yet remaining long enough to allow cytoplasmic polyadenylation. Therefore, dysregulation or inhibition of PARN alters the balance, permitting excessive poly (A) tail elongation by cytoplasmic polyadenylation and influencing translational activation. This mechanism is relevant for the later discussion on the regulation of telomerase‐associated RNAs such as TERC and TERRA. Thus, as shown in Figure 1, the length of the poly (A) tail by polyadenylation or deadenylation is a rate‐limiting process in maintaining RNA stability, which in turn regulates gene expression.

### 2.3 The TERC and PARN Connection

**FIGURE 2:**
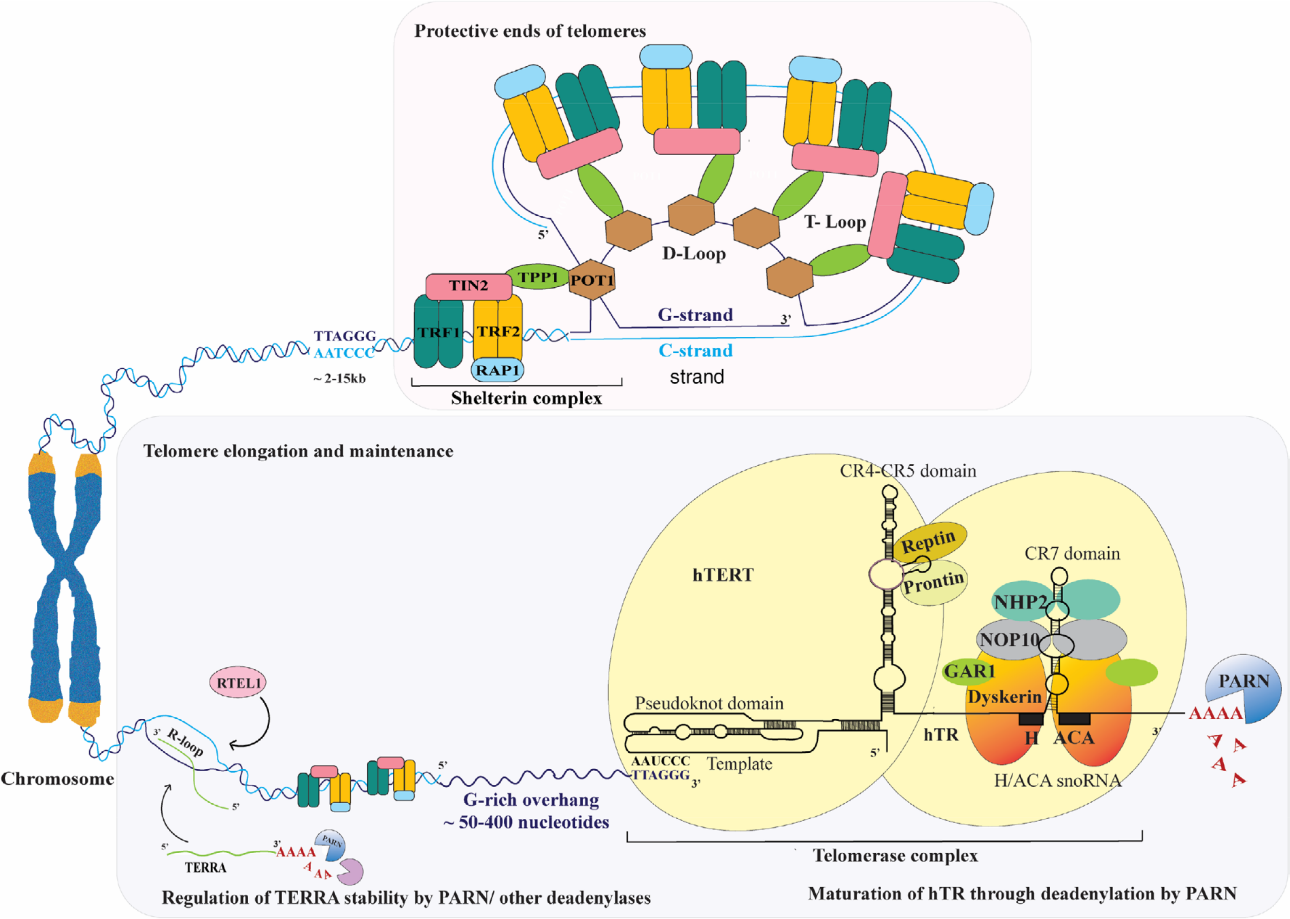
Schematic representation of PARN's role in telomere length maintenance, illustrating its interactions with TERC. The above figure depicts a chromosome that has a protective cap structure called telomeres made up of a sequentially repeated hexanucleotide sequences “TTAGGG.” In humans, telomere length can range from approximately 15 kb at birth, which can overtime shorten with each cell division reaching a critical length of 2 kb, triggering senescence and apoptosis. They are maintained by two main ribonucleoprotein complexes: The telomerase complex (TERC/hTR, TERT/hTERT and Dyskerin) and the Shelterin complex. The telomerase complex comprises a holoenzyme consisting of two lobes tethered to the RNA component of telomeres (TERC/hTR), comprising two domains: A template/pseudoknot domain (t/PK) and the conserved regions 4 and 5 (CR4/5) that interacts with the catalytic subunit telomerase reverse transcriptase (TERT/hTERT). The opposite lobe of the telomerase holoenzyme contains hTR's 3′ portion, that have a small nucleolar RNA (snoRNAs) motif called an H/ACA box, which is linked to a dimer of proteins forming ribonucleoprotein (RNP) complexes. These RNP complexes are localized in the nucleus, which promotes telomere biogenesis. There are four core H/ACA proteins that include two Dyskerin molecules and NHP2 bound to hTR through H and ACA boxes. Whereas GAR1 and NOP10 are bound to Dyskerin. TERT with Dyskerin forms complexes with two seldom recognized ATPases called Prontin and Reptin, which enhance the telomerase assembly. The second complex, called the Shelterin complex, consists of six proteins, namely, TRF1/TERF1 and TRF2/TERF2 that binds directly to the telomeric; POT1, which binds specifically to the 3′ single‐stranded G‐overhang; TPP1, which recruits and stabilizes POT1 on the teloemric DNA; TIN2 that establishes a bridge between the telomeric DNA‐binding proteins and those attached to the single‐sranded telomeric overhang through its interaction with both TRF1 and TRF2; and the repressor or activator protein (RAP1) that coats telomeric DNA and aids in protecting its ends from nuclease digestion, by localizing with telomeres through its association with TRF2. Together, these Shelterin complex attach onto the telomeric ends where telomere sequence terminates with a single‐stranded G‐rich overhang (50–400 nucleotides), which protrudes at the 3′ end. This extension then loops back to create a telomeric loop (T‐loop) and invades 5′ double‐stranded telomeric duplex, forming a structure known as displacement loop (D‐loop). This creates a distinctive lariat shape that protects the 3′ OH end of the DNA from being recognized as a double‐stranded break, thereby sheltering it from DNA damage repair mechanisms. When combined, they provide a framework that guards against chromosomal fusion and breakdown. PARN contributes to telomere maintenance through its involvement in the maturation of TERC by deadenylating its poly (A) tail. Dyskerin then binds to the mature TERC with other H/ACA proteins activating the telomerase mechanism. TERRA is a long noncoding RNA transcribed from telomere repeats and is involved in the maintenance of telomere length. In its non‐polyadenylated state, it in forms RNA–DNA hybrids (R‐loop) by base pairing with telomeres and contributes to telomere stability by the ALT pathway. RTEL1 a DNA helicase plays a critical role in resolving secondary DNA structures in telomere ends, such as T‐loops and G‐quadraplexes during DNA replication, and also associates with TERRA in resolving R‐loops, thereby preventing telomere loss. This image was created using adobe illustrator.

Variations in genes involved in telomere maintenance, including *DKC1*, which codes for a nucleolar protein (dyskerin) that is essential for telomerase RNA stability, *PARN*, which codes for a ribonuclease involved in maturation and stability of RNA, *NOP10* and *NHP2* : encodes crucial subunits of the H/ACA ribonucleoprotein complex required for telomerase assembly; and *TERC*: a noncoding telomerase RNA component that is the essential RNA template for the telomerase to add DNA repeats to the telomeres, have been found to affect telomere biology in a cell (Figure 2). As discussed above, PARN is traditionally known to be involved in the first step of mRNA degradation by deadenylation. The first evidence of PARN's role in posttranscriptional maturation of TERC, was shown in 2015, when pathogenic variations in the *PARN* gene were identified in dyskeratosis congenita (DC) patients. In the investigation that followed, it became clear that insufficient levels of PARN in DC patient cells resulted in the accumulation of immature TERC forms, which in turn causes dysregulated telomerase function (Moon et al. 2015). This finding led to the notion that PARN, which removes poly (A) residues in mRNAs initiating mRNA decay, has a similar role in trimming poly (A) residues of TERC, a step required for its maturation. As explained above, it has been clear that PARN and PAPD5 both play a role in tailoring the ends of the 3′ poly (A) tail length of RNA. In further efforts toward developing therapeutic strategies to reverse the effects of telomere disorder caused by PARN deficiency, an inhibitor of poly (A) polymerase (PAPD5), targeted against the TERC poly‐A tail, was devised (Boyraz et al. 2016; Nagpal et al. 2020). The resulting evidence demonstrated an increased level of mature and steady‐state TERC and telomerase activity, suggesting opposing feedback between the two factors. This study further suggested that regardless of the presence of many other deadenylases and poly (A) polymerases, PARN and PAPD5 are nonredundant when it comes to TERC maturation. However, from a therapeutic point of view, the likely off‐target of the PAPD5 inhibitor needs to be taken into account, as PAPD5 is also involved in the regulation of other cellular RNA species (Rammelt et al. 2011; Berndt et al. 2012; Boele et al. 2014). A global transcriptome analysis by Boyraz et al. revealed that PAPD5 inhibition in patient iPSC cells did not affect the normal cellular viability. About 11 genes were deregulated in PAPD5‐deficient cells, which were only the noncoding RNAs, especially snoRNAs. Similarly, Nagpal et al., in their study on patient‐derived induced pluripotent stem cells (iPSCs), reported that PAPD5 inhibition did not result in cellular toxicity, suggesting no effects on cell viability. However, both these studies did not look at the long‐term effects of PAPD5 inhibition to clearly define the extent and the severity of the likely off‐target effects. Therefore, long‐term studies are necessary to ascertain the suitability of PAPD5 as a therapeutic agent in a clinical setting.

Nevertheless, these observations illustrated the significance of the involvement of PARN and PAPD5 in TERC function, and they further validated the potential of novel clinical interventions that target the PARN–PAPD5 loop in TBD management (Kam et al. 2021). Similarly, inhibition of TERC deregulation by RNA‐degrading exonucleases could also prove to be an alternative strategy to treat telomere disorders by understanding the crosstalk between three genes, namely *PARN*, Target of Egr1 (*TOE1*), and dyskerin (*DKC1*). It has been shown that inefficient binding of DKC1 to TERC results in its degradation by tail and cap‐degrading exonucleases (Zhang et al. 1999). The presence of PARN limits this effect by deadenylating TERC. TOE1, which is also a 3′‐end exonuclease, that exhibits deadenylation as well as decay of non‐poly (A) sequences of RNA, functions redundantly with PARN in enabling maturation and stability of noncoding RNAs, including the telomerase RNA. Disruptions of this process have been known to cause pontocerebellar hypoplasia 7 (PHC7), a type of neurodevelopmental disorder (Huynh and Parker 2023). This highlighted the specialized activity of PARN, which does not merely act as a general 3′‐5′ exonuclease removing poly(A) tails: rather, it regulates poly(A) tail length with PAPD5, generating transcripts with optimized tail length depending on the transcript identity and cellular conditions, thereby implying the importance of controlled RNA 3′‐end trimming in telomere biology. These observations implied that the telomeres get deregulated due to the loss of these regulatory genes, likely due to degradation or accumulation of TERC. This pathway was supported by studies involving knockdown of RNA decay factors such as mRNA decapping enzyme 2 (DCP2), which encodes for a protein that initiates 5′‐3′ degradation of mRNA by removing the 7‐methylguanosine cap, and exosome component 10 (EXOC10), a catalytic subunit of nuclear RNA exosome, that is involved in 3′‐5′ RNA degradation. Suppression of these exonucleases restored TERC levels by limiting RNA degradation, compensating for the defective PARN as seen in TBD patients, suggesting a useful strategy in developing clinical targets to treat TBDs (Shukla et al. 2016; Deng et al. 2019). Thus, it is now clear that variations in *PARN* and other genes that code for proteins implicated in telomere‐binding and TERC/hTR (human telomerase RNA) processing reduce the levels of TERC, which result in a wide spectrum of TBDs.

### 2.4 The Terra and PARN Connection

A number of seminal studies have made it clear that the process of telomere shortening beyond the threshold length results in telomere decapping, which refers to the loss of the protective proteins cap (Shelterin complex), resulting in the disruption of the stable t‐loop structure at chromosome ends (Figure 2). This leads to telomeric DNA being incorrectly exposed as a DNA double strand break, which in turn triggers a DNA damage response, subsequently leading to an arrest in the cell cycle (Klauser et al. 2021). To avoid telomere malfunction and to encourage replication stress at telomeres, telomeric repeat‐containing RNA (TERRA), a long noncoding RNA transcribed from telomeric repeats, is also present (Figure 2). Studies involving fluorescence in situ hybridization and cellular fractionation observed an enriched presence of TERRA transcripts in human telomeres (Savoca et al. 2023). While its biogenesis is not fully understood, approximately 10% of TERRA transcripts are in polyadenylated form in a telomere‐specific manner and are predominantly found to be located in the nucleoplasmic region where they are not associated with telomeres, whereas non‐polyadenylated TERRA transcripts are chromatin associated in the nucleoplasm (Porro et al. 2010). These transcripts tend to co‐localize with telomerase to regulate telomere length (Bettin et al. 2024). Functionally, TERRA contributes to telomere protection by holding sister telomeres together via RNA–DNA hybrid complexes where TERRA RNA forms sequence homology with telomeric DNA, giving rise to R‐loops (Figure 2). These structures promote telomere elongation linked to the alternative lengthening of telomere (ALT) pathway, which is predominantly activated in cancer cells (Roychoudhury et al. 2020; Fernandes et al. 2021). PARN is known to regulate TERRA RNA stability by modulating its poly (A) tail, contributing to telomere length maintenance (Tummala et al. 2015). However, it is important to note that PARN is not the sole regulatory factor controlling TERRA's stability; in its absence or reduced expression, regulation of TERRA poly(A) tail may proceed through other deadenylases or exosome‐mediated RNA decay pathways (Savoca et al. 2023). Depletion of TERRA causes the loss of sister telomere cohesion, resulting in the disruption of R‐loops, contributing to deregulated telomeres (Zeinoun et al. 2023; Sze et al. 2023; Azzalin 2025). Taken together, these observations further expand the role of PARN as not just being a 3′‐5′ exonuclease but also as one of the regulators of telomere biology, wherein PARN‐TERC directly promotes telomere lengthening, whereas PARN‐TERRA regulates telomere homeostasis, through a regulatory mechanism that doesn't involve degradation of TERRA transcript. Thus, this functional crosstalk between TERC‐PARN and TERRA‐PARN offers a deeper mechanistic insight into telomere maintenance pathway and may serve as a potential treatment strategy for TBDs.

## 3 Emerging Evidence of PARN'S Role in TBDs: From in Vitro Studies to Clinical Findings

The telomere length is maintained by the association between its RNA component and multiple proteins attached to it. Intriguingly, recent studies have shown that PARN, a deadenylase enzyme, is also involved in this process. Hence, any impairment in the interaction of these proteins or enzymes is likely to result in inadequate telomerase activity, a common molecular manifestation in a group of clinical conditions called TBDs (Lima et al. 2017). This section discusses the participation of PARN in the maintenance of telomere stability and how it is perturbed in patients with *PARN* variants. Thus, understanding the interplay of these two tail modifiers in the context of diseases becomes important in the development of targeted therapy for such rare conditions.

Considering the role of PARN in pathways that regulate the telomere RNA component, the identification of pathogenic variants in *PARN* in TBD patients was hardly a surprise. One of the first reports that truly implicated PARN in TBDs was the discovery of biallelic mutations in the *PARN* gene in three families with severe DC (Rivosecchi et al. 2024). These variations impacted the critical domains and decreased the deadenylation activity of PARN, which resulted in a reduction in cell viability, an early p53‐dependent DNA damage response, and reduced transcript levels of telomere biology genes such as *TERC, DKC1, RTEL1*. The functions of TERC and DKC1 have been described in the introduction section. RTEL1 is a DNA helicase involved in telomere replication and genome stability. It plays a critical role in resolving secondary DNA structures in telomere ends, such as T‐loops and G‐quadruplexes during DNA replication and is also associated with TERRA in resolving R‐loops thereby preventing telomere loss. TERF1/TRF1 are components of the shelterin complex that regulates the length and stability of telomere (Dodson et al. 2019). Figure 2 illustrates their molecular relationship and summarizes the interconnected pathways that demonstrate the role of PARN in telomere maintenance. Earlier studies also reported monoallelic mutations in *PARN* in patients with severe bone marrow failure, mental illness, developmental delays, and central hypomyelination (Dhanraj et al. 2015). Additionally, short telomeres, an abnormal ribosome profile, and poor oligoadenylation of particular H/ACA box snoRNAs were seen in the patient‐derived PARN‐deficient cells. The causative relationship was further validated by PARN loss‐of‐function studies using zebrafish and human marrow cells (Benyelles et al. 2019). Similarly, novel biallelic variants in the *PARN*, including a putative regulatory variation in the 5′ UTR, were found in a patient with autosomal recessive Hoyeraal‐Hreidarsson syndrome (HHS), a severe form of DK. These pathogenic variations resulted in a negative impact on telomere biology by interfering with its RNA stability, triggering abnormal DNA damage response and causing downregulation of telomere biology proteins (Burris et al. 2016). When the whole exome sequence of a neurodevelopmental disorder patient, having short telomeres, was analyzed, pathogenic heterozygous variants in the *PARN* gene were identified (Deng et al. 2019). In another instance, a genetic investigation of an interstitial lung disease (ILD) patient revealed the presence of a heterozygous splice acceptor variant in the *PARN* gene (Ketharnathan et al. 2023). Further investigation carried out by screening young individuals with a family having clustered hematologic abnormalities and who were also highly susceptible to ILD revealed 17 heterozygous *PARN* variant carriers. Given the fact that telomere function is perturbed in PARN‐deficient cells, providing clinical vigilance to track its effect on telomeres will help in understanding the severity of the disease (Macko et al. 2023). Taken together, these findings strongly support the role of PARN in TBDs, and a thorough investigation of this new finding could potentially help in developing targeted therapeutic strategies for such rare conditions (Table 1).

**TABLE 1:** Allelic variants of the *PARN* gene in telomere biology disorders (TBDs).

Sl. No.	Telomere biology disorder phenotype	*PARN* variant (HGVS/SCV ID)	Mutation type	Functional effects	Clinical manifestations	References
1	Dyskeratosis congenita, autosomal recessive (Chromosome 16p13.12)	NM_002582.4(PARN):c.1148C>T (p.Ala383Val)/SCV000206797	Homozygous missense	A pathogenic PARN variant protein displayed decreased deadenylation activity accompanied by cell cycle arrest at the G2/M phase and increased cell death especially after UV‐induced DNA damage.	Bone marrow failure with the depletion of CD34+ hematopoietic progenitor cells, abnormal skin pigmentation, nail dystrophy, oral leukoplakia, intrauterine growth retardation, microcephaly, cerebellar hypoplasia, developmental delay, sparse hair, and dental caries.	Tummala et al. (2015), Dhanraj et al. (2015)
		NM_002582.4(PARN):c.918 + 1G>T/SCV000206798	Homozygous splice‐site	This variant affected a donor splice site in intron 13 of the PARN gene, disrupting RNA splicing, leading to a loss of protein function.		
		NM_002582.4(PARN):c.863dup (p.Asn288fs)/SCV000206799	Compound heterozygous mutations in the *PARN* gene: a 1‐bp duplication, resulting in a frameshift and premature termination; second allele with a 4‐bp deletion in intron 9, predicted to result in abnormal splicing, exon skipping, and an in‐frame deletion in the R3H domain	A 1 bp duplication resulted in a frameshift mutation and premature termination. The second allele with a 4 bp deletion predicted to result in abnormal splicing, exon skipping, and an in‐frame deletion in the R3H domain responsible for poly (A) tail binding.		
		NM_002582.4 (PARN):c.659+ 4_659+7del	PARN, 4‐BP DEL, 659AGTA; 4‐bp deletion in intron 9 of the PARN gene that was found in a compound heterozygous state	This particular pathogenic variant disrupts the consensus splice site, causing aberrant splicing, reported in individuals with bone marrow failure disorders.		
		NM_002582.4 (PARN):c.1045C>T (p.Arg349Trp)	Compound heterozygous mutations in the PARN gene: a c.1045C‐T transition in exon 16, resulting in an arg349‐to‐trp (R349W) substitution in the nuclease domain.	Alteration in the nuclease domain is associated with impaired PARN function and defective telomere maintenance, although functional evidence remains limited.		
		NM_002582.3(PARN):c.962 + 295_1263‐8706del	PARN, 22‐KB deletion	A pathogenic variant with a neurologic impairment consistent with a diagnosis of Hoyeraal‐Hreidarsson syndrome.		
2.	Pulmonary fibrosis and/or bone marrow failure syndrome, telomere‐related,4, autosomal dominant (Chromosomal location: 16p13.12)	NM_002582.4(PARN):c.246‐2A>G	Heterozygous A‐to‐G transition in intron 4 of the *PARN* gene	This pathogenic variant showed a telomere length less than 1% of control length, consistent with telomere dysfunction.	Premature graying of the hair, lung fibrosis, a high prevalence of squamous cell carcinomas, particularly of the head and neck, anus, and skin, predominantly in males, with tumor development linked to CD4^+^ T‐cell lymphopenia, impaired immune surveillance, and, in some cases, HPV infection or secondary immunosuppressive conditions.	Stuart et al. (2015); Schratz et al. (2023)
		NM_002582.4(PARN):c.529C>T (p.Gln177Ter)	Heterozygous c.529C‐T transition in the *PARN* gene, resulting in a gln177‐to‐ter (Q177X) substitution in the CAF1 ribonuclease domain.	This is a pathogenic PARN variant observed telomere length in the proband was less than 1% of control length.		
		NM_002582.4(PARN):c.563dup (p.Glu189fs)	Heterozygous 1‐bp insertion in the *PARN* gene, resulting in a frameshift and premature termination (Ile188IlefsTer7) in the CAF1 ribonuclease domain.	This pathogenic variant, resulted in a frameshift mutation and premature termination codon in the CAF1 ribonuclease domain, leading to reduced telomere length observed in the proband, which was less than 7% of control length.		
		NM_002582.4(PARN):c.1262A>G (p.Lys421Arg)	Heterozygous c.1262A‐G transition in the *PARN* gene, resulting in a lys421‐to‐arg (K421R) substitution at a highly conserved residue.	This pathogenic variant was also associated with telomere length shortening, which was less than 1% of control length, consistent with telomere dysfunction.		

*Source:* https://www.omim.org/allelicVariants/604212, https://www.ncbi.nlm.nih.gov/clinvar/.

## 4 The Interplay of PARN and Telomerase in Cancer: p53 in the Loop?

**FIGURE 3:**
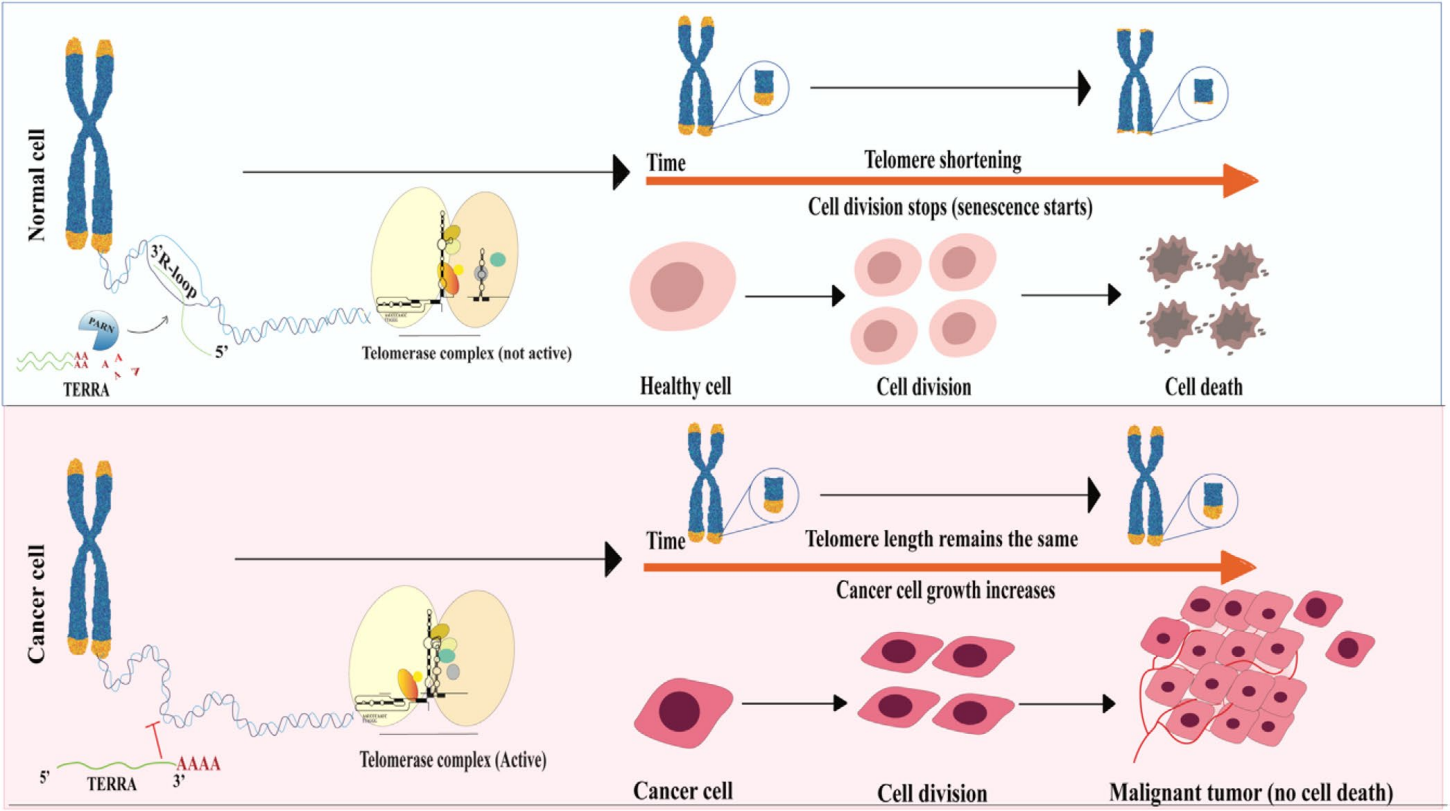
Illustration of varying telomere length in non‐neoplastic and neoplastic cells. The telomere dynamics in normal cells and cancer cells are shown in this figure. In normal somatic cells, progressive telomere shortening activates DNA damage responses that induce senescence or apoptosis as a protective mechanism. Non‐polyadenylated TERRA transcripts localize near the telomeres and contribute to telomere homeostasis. Biogenesis of TERRA occurs independently of PARN; however, PARN regulates stability and turnover of polyadenylated TERRA transcripts. In contrast, in cancer, telomerase reactivation stabilizes short telomeres, encouraging cellular immortalization and malignant behavior. This image was created using Adobe Illustrator.

**FIGURE 4:**
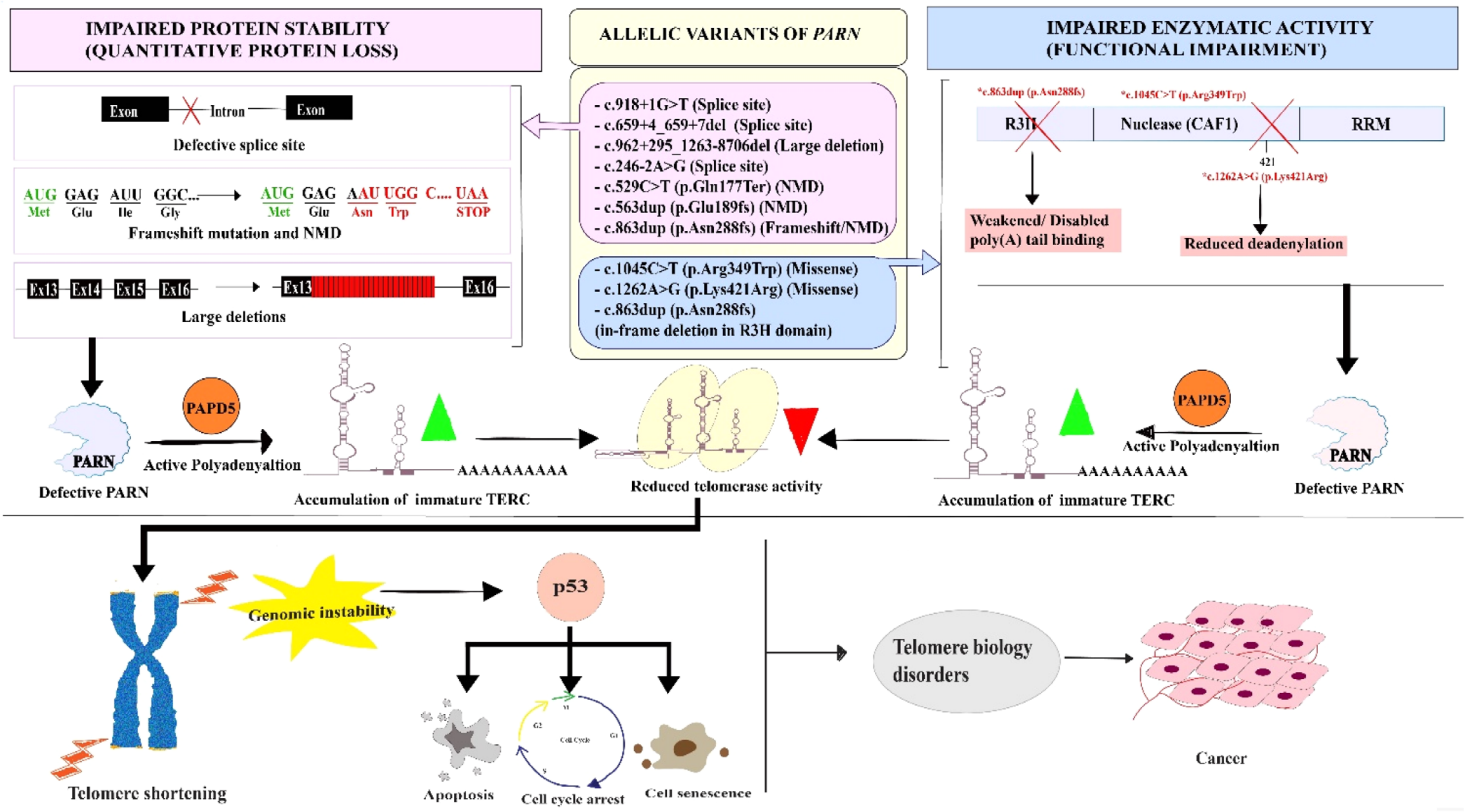
Depiction of the Interplay between PARN, telomere maintenance, and p53 signaling. The above figure illustrates how the identified *PARN* genetic variants drive clinical pathogenesis, in telomerase deregulation, with p53 involvement contributing to a high cancer risk condition in TBD patients. The identified *PARN* variants are displayed in the diagram along with their variation status. The quantitative loss of PARN protein due to impaired protein stability (Top left, pink) occurs as a result of defective splice sites, frame shift mutations, and nonsense‐mediated decay (NMD). Conversely, functional/enzymatic impairment despite stable protein abundance (Top right, blue) is displayed as a consequence of missense mutations or a disabled R3H domain, which is primarily involved in poly (A) tail binding. Usually, PARN promotes TERC maturation by removing PAPD5‐mediated oligoadenylation, which prevents RNA decay. The loss of functional PARN leaves PAPD5 unopposed, causing hyper‐polyadenylation resulting in accumulation of polyadenylated TERC (immature TERC shown in green cone) leading to its decay. This leads to lack of telomerase activity (shown in red cone). A reduced telomerase activity triggers genomic destabilization through shortening of telomeres at the end of the chromosomes. This recruits DNA damage signaling, phosphorylating p53, which then induces DDR that halt the cell cycle process, thereby promoting apoptosis or cell senescence. This results in severe clinical phenotypes as observed in TBD patients with skin aberrations, nail dysplasia and others. Over time, chromosomal fusions tend to progress into cancer, which decreases the patient's survival rate. This image was created using Adobe Illustrator.

An increase in replication stress can result in DNA damage and genomic instability, which could enhance the risk of developing neoplastic conditions (Figure 3). As discussed above, patients with DC that harbor variations in genes involved in telomere maintenance, including *PARN*, also have a high risk of developing cancer (Table 2). Thus, it is important to understand how the interplay between these two tail modifiers has consequences beyond telomere deregulation. Variations in the *PARN* and other telomere maintenance genes, including *TERT*, *TERC*, and *RTEL1*, were initially discovered in pulmonary fibrosis and DC patients (Feurstein et al. 2020; Xing and Garcia 2016). These patients carried short telomeres, as evident in leukocyte telomere length assays. Interestingly, p53 mutant mice that overexpress mutated p53 and show an impaired MDM2 regulation also displayed these defects in telomere maintenance and DNA repair, resembling symptoms of DC and Fanconi anemia. These defects in telomere maintenance arising through telomere decapping result in the formation of telomere‐dysfunction‐induced foci composed of DNA damage proteins at the decapped telomere, which, through Ataxia telangiectasia mutated (ATM) and ATM and RAD3‐related (ATR) kinases, phosphorylates p53, inhibiting its interaction with MDM2, thereby elevating the levels of p53. This further transcriptionally upregulates target genes that mediate cell‐cycle arrest, senescence, and apoptosis. In this mouse model, the hyperactivation of p53 arose from the loss of carboxyl terminal domain (CTD), which is a basic disordered regulatory region that modulates DNA binding, interacts with MDM2, and is also crucial for p53's tumor suppressor functions, while p53's intrinsically disordered N‐terminal domain (NTD) acts as a transcription activator, targeting specific genes, which is fine‐tuned through posttranslational modification and non‐specific DNA interactions by CTD ensuring proper cellular response to stress. Loss of the CTD region, as demonstrated in the p53 variant mice, resulted in a hyperactive p53, which has a deregulated transcriptional function. As a result, it not only enhanced DNA damage response pathways but also deregulated telomere pathways by repressing genes in telomere maintenance, such as components of the shelterin complex (*TERF1* and *TINF2*) and telomerase (*DKC1*). Other p53‐target genes that encoded for telomere maintenance were *GAR1* (Figure 2) (Toufektchan and Toledo 2018). Together, these findings suggest a potential link between hyperactive p53 signaling, decapped telomeres, defective DNA repair, and accelerated aging. Indeed, based on the studies in p53 mutant mice, it was suggested that polymorphisms affecting the p53 pathway might play the role of genetic modifiers of telomere biology and bone marrow failure in TBD patients. In line with this hypothesis, a germline missense *MDM4* mutation was discovered in a family exhibiting DC‐like manifestations that included short telomeres, bone marrow failure, and acute myeloid leukemia. The presence of this pathogenic variation in *MDM4* resulted in an enhanced p53 activity due to the disruption of the MDM2‐p53 feedback loop, consequently leading to telomere shortening (Toufektchan et al. 2020; Maragozidis et al. 2012). Similarly, in PARN‐deficient and knockout models associated with HHS, shelterin genes of the telomeres display destabilization, along with defects in ribosomal RNA biosynthesis. Additionally, it has been shown that reduced levels of DKC1, a core protein that binds to activate telomerase, have been shown to regulate p53 activity at the level of mRNA translation. In instances of impaired dyskerin function, inactivation of p53 was observed, whereas activation of p53 resulted in the downregulation of *DKC1* and other telomere maintenance genes, revealing a regulatory loop between cancer and telomere biology (Montanaro et al. 2010). The role of microRNAs (miRNAs) in modulating the expression of the *TP53* gene was also studied in the context of telomere regulation. For instance, in the year 2019, Shukla et al. developed a patentable innovation of administering a PARN inhibitor in addition to a chemotherapeutic drug to treat cancer. This discovery was based on their research findings, which demonstrated the protective role of PARN in miRNAs by deadenylating them to avoid being oligoadenylated by PAPD5, which otherwise would ensure their degradation by 3′ to 5′ DISL and DIS3L exonucleases. In the same study, it was further shown that PARN deficiency led to a reduction in the stability of miRNAs, which in turn resulted in the accumulation of TP53 mRNA levels in PARN‐deficient patients. Hence, these findings highlight PARN as a potential therapeutic target in chemotherapeutic‐resistant cancer treatment; however, PARN inhibitors remain in experimental stages, and no approved clinical therapies are currently available (Shukla, Bjerke, et al. 2019; Shukla, Spurrier, et al. 2019). An investigation involving a telomerase‐deficient zebrafish model revealed features of premature infertility and early mortality in the mutants, along with enhanced DNA damage and poor cell proliferation. The study further progressed to investigate a cross between p53 mutant zebrafish carrying p53M214K mutation located in the DNA binding domain (regulated by CTD), which affects the transcriptional activation of target genes (regulated by NTD), with telomerase‐deficient zebrafish carrying *tert*hu340 mutation, which impairs telomerase activity. The progeny presented partial recovery of the lifespan and fertility of male zebrafish by reversing their early aging phenotype. This reciprocal recovery of the p53 mutant phenotype in telomerase‐deficient zebrafish suggested a complicated functional relationship between p53 signaling, telomere maintenance, aging, and cancer (Şerifoğlu et al. 2024). On the other hand, as discussed in the earlier sections, PARN and p53 participate in a negative regulatory feedback loop. Under non‐stress conditions, PARN activity keeps p53 expression levels low by controlling miRNA biogenesis and TP53 mRNA stability. Upon DNA damage, an increase in p53 activates PARN deadenylase in a transactivation‐independent manner, thereby regulating gene expression during DNA damage response and functionally linking 3′‐end processing with p53 signaling and genome stability pathways (Murphy and Kleiman 2020). Surprisingly, telomerase activity within mononuclear cells was found to be regulated by gonadal steroids, especially estrogen (Benko et al. 2012). Besides, PARN‐deficient zebrafish models presented compromised gonadal maturation in males, suggesting its critical role in oogenesis (Nanjappa et al. 2023; Felicitus et al. 2024). This brings about queries concerning gender‐specific functions of PARN and its impact on telomerase activity. An interesting suggestion based on the previous discussions would be to explore whether PARN could play a major role in this nexus (Figure 4).

**TABLE 2:** Differential PARN expression and its biological consequences in cancer and other human diseases.

Event/disease	PARN expression	Its effect on other processes or genes	Mechanism/role	Future therapeutic strategies/interventions	References
Breast cancer	Downregulated	Upregulation of *PLD1*	Depletion of PARN increased ARE‐mediated stability of PLD1 levels, which in turn aggravated the proliferation of breast cancer cells.	PARN can serve as a potential biomarker or a therapeutic target	Miller (2017)
Acute myeloid leukemia and Acute lymphoblastic leukemia (AML, ALL)	Upregulated	Increased degradation of mRNAs in leukemia	Increased phosphorylation of PARN was observed which enhanced their deadenylation activity on other mRNAs in leukemic cells.	PARN can serve as a biomarker or a therapeutic target	Maragozidis et al. (2012)
Human colon cancer cell lines (HCT 116)	Upregulated	Decreased in TP53 mRNA expression	The presence of ARE‐and miRNA regions in the 3′ UTR of regions in TP53 mRNA promoted PARN‐mediated decay.	PARN inhibitors can help restore p53 levels and may help manage cancer	Zhang et al. (2015)
Squamous cell lung cancer (SCC)	Increased	The degradation of transcripts responsible for cell adhesion and proliferation	Increased expression of PARN encouraged the degradation of mRNAs having metastatic and proliferative tendencies, which correlated with the increased survival rate of patients. Whereas experimental knockdown of *PARN* induced variation in these genes.	Further research on PARN and its role in this particular cancer is necessary, which can help in understand its therapeutic application.	Maragozidis et al. (2015)
Bioinformatics analysis of SCC					
NCI‐H 520 cell lines	Experimental knockdown of *PARN*	Variation of genes related to cell adhesion, cell junction, and metabolism			
Gastric cancer	Upregulation of *PARN*	Increased proliferation, no observations on other genes were made	Proliferation of cancer cells was observed, with a cell‐ type‐ dependent RNA stability regulation.	Different types of cells react differently to stress, giving a differential expression of PARN in each cell type. Therefore, further analysis is required.	Zhang et al. (2015)
MKN28 and AGS gastric cancer cell lines	Experimental knockdown of *PARN*	Upregulation of p53 and p21 resulted in arrest at the G0/G1 phase of the cell cycle	Proliferation of these cancer cells were inhibited and p21 at 3′ UTR triggered the action of PARN in AGS cells only.		
Developmental/mental illness, Bone marrow failure, and central hypomyelination	Monoallelic mutations of *PARN*	Cells from this patient had impaired oligoadenylation of specific H/ACA box snoRNAs.	PARN‐deficient patient cells manifested short telomeres and an aberrant ribosome profile similar to those described in some variants of dyskeratosis congenital.	*PARN* mutations manifested a severe form of dyskeratosis congenita, whereas PARN deficiency showed short telomeres due to instability of telomerase RNA.	Dhanraj et al. (2015)
	Biallelic mutations of *PARN*				
	An additional missense mutation on the non‐deleted allele severely reduced PARN protein and deadenylation activity.				
Alzheimer's disease	PARN, Tau, and Pin1 (mitotic regulator overexpressed in cancer but depleted in Alzheimer's disease)	Tau protein forms complexes with p53 and PARN	Tau induces PARN activity during DNA damage response, increased by p53 involvement.	Potential neurodegeneration therapeutic target.	Baquero et al. (2019)
Solid tumors and hematopoietic malignancy	Downregulation of *PARN*	No observations were made	Tumor suppressive function of PARN may be suggested	Further screening of a large number of samples would be required to evaluate the speculated potential of PARN as a novel biomarker in solid organ and hematopoietic cancer.	Babu et al. (2021)
Lung cancer cells NCIH‐460 and NCIH‐522	Experimental knockdown of *PARN*	Downregulation of tumor suppressor genes and inconsistent downregulation of oncogenes in lung cancer cells	Cell‐specific alteration in the expression of tumor suppressor and oncogenes on PARN depletion	Since PARN is dependent on the cell type for its expression, further comprehensive research is warranted to elucidate the precise mechanisms underlying PARN's involvement in lung cancer pathogenesis, thus paving the way for more targeted therapeutic strategies	Babu et al. (2022)
Bioinformatic analysis (gastric, head and neck, squamous cell carcinomas)	Upregulated *PARN* expression	Inhibited apoptosis	PARN has an anti‐apoptotic effect that also helps in promoting the proliferation and migration of these cancer cells	A potential target for diagnosis, prognosis, and therapeutic strategy	Zhang et al. (2023)
Esophageal cancer (EC) tissues					
Esophageal cancer cells	Experimental knockout of *PARN*	Upregulation of BIM, IGFBP‐5, and p21 levels Downregulation of survivin and sTNFR‐2 (antiapoptotic proteins), Akt, p‐Akt, PIK3CA, and CCND1 in the downstream signaling pathway in the regulation of EC progression	Increased rate of apoptosis thus inhibiting the proliferation and metastatic abilities of tumor cells.		
Glioblastoma	Upregulated *PARN* expression	Increased expression of EGFR activating STAT3 (plays a role in gliomagenesis)	Resulted in the destruction of the EGFR‐targeting miRNA miR‐7 increases the proliferation of glioblastoma cells.	Potentially offering new avenues for therapeutic intervention in this glioblastoma.	Yin et al. (2023)
PARN knockdown in xenograft and Glioblastoma stem cells (GSCs)	Depletion of *PARN*	Reduced expression of *EGFR*	Increased survival rates of glioblastoma disease models		

## 5 Conclusion

It is now quite clear that the stability of a genome is maintained by the complex interaction between PARN and the telomerase enzyme. Where telomerase protects the chromosomal ends from fusion and also enhances cell replication, PARN, on the other hand, plays a role in the maturation of telomerase RNA and controls noncoding RNAs like TERRA, which modulates telomerase activity. Introduction of p53 in the interplay adds another level of complexity, linking telomere dynamics to the responses to DNA damage and cell fate. It has been amply demonstrated that interruptions in any of these processes can contribute to a variety of human diseases, ranging from TBDs to cancer. Nevertheless, how PARN deficiency affects p53 signaling and the potential for gender‐specific effects observed in zebrafish mutant models still remain unclear. Addressing these unanswered questions will help convert molecular findings into focused treatment approaches.

## Author Contributions

**Shalon Suzanne Pinto:** writing – original draft, investigation, conceptualization, methodology, visualization, data curation, writing – review and editing. **Sujitha Felicitus:** writing – original draft, investigation, conceptualization, methodology, visualization, data curation, writing – review and editing. **Sai Samanvitha Madagundapalli Ramakrishna:** investigation, methodology, visualization, writing – review and editing. **Dechamma Pandyanada Nanjappa:** writing – review and editing, investigation, visualization, conceptualization, validation. **Gunimala Chakraborty:** writing – review and editing, resources, validation, funding acquisition. **Anirban Chakraborty:** conceptualization, investigation, funding acquisition, visualization, validation, writing – review and editing, supervision, resources, writing – original draft.

## Funding

The authors have nothing to report.

## Conflicts of Interest

The authors declare no conflicts of interest.

## Data Availability

Data sharing is not applicable to this article as no new data were created or analyzed in this study.
